# Learning Communities Engage Medical Students: A COVID-19 Virtual Conversation Series

**DOI:** 10.7759/cureus.9593

**Published:** 2020-08-06

**Authors:** Emilyn Anderi, LaToya Sherman, Sara Saymuah, Eric Ayers, Heidi T Kromrei

**Affiliations:** 1 Office of Student Affairs, Wayne State University School of Medicine, Detroit, USA; 2 Office of Learning and Teaching, Wayne State University School of Medicine, Detroit, USA; 3 Internal Medicine/Pediatrics, Wayne State University, Detroit, USA

**Keywords:** learning communities, covid-19, medical students, zoom, virtual, engagement

## Abstract

Context

Challenges to medical education have been pervasive during the COVID-19 pandemic, and medical students, in particular, have faced numerous obstacles as a result. One of the greatest losses for medical students was the inability to gather with their peers and a lost sense of community. The Learning Community (LC) program at Wayne State University School of Medicine (WSU SoM) expanded our offerings through the use of the Zoom platform to increase a sense of connectedness among medical students. The first initiative of its kind at WSU SoM, the Virtual Conversation series enabled students to share their pandemic challenges while also connecting with physicians on the COVID-19 frontlines.

Students were offered eight online sessions with physicians and residents who were able to share insight regarding (1) how to succeed as a medical student on rotation during COVID-19, (2) potential implications of the pandemic on residency applications, (3) the utility of telemedicine, (4) tips for patient encounters, and (5) realities of serving as a physician during a global health crisis.

Methods

Residents and clinical physicians on the COVID-19 frontlines participated in 40-minute discussions with WSU SoM students through Zoom. Electronic Qualtrics surveys were distributed to medical student attendees of the Virtual Conversation series and responses were received via Likert scale, open text, and ranking questions.

Results

Qualtrics results demonstrated 55% of medical students (n=55) reported they learned new information about the COVID-19 pandemic from the perspective of physicians. Additionally, 62% of medical students described the Virtual Conversation series as ‘extremely useful’.

Conclusion

The Virtual Conversation series emphasizing different medical aspects of COVID-19 provided a unique benefit to medical students’ understanding of the current landscape of healthcare, the anticipation of their future roles as physicians, connectedness with their community, and opportunity to practice flexibility as they begin to apply online learning with real-world situations in the health system.

## Introduction

Remote learning has emerged to the forefront of academic content delivery, posing a unique challenge to building community and rapport among peers in academic programs. Over the past few years, medical schools have been moving away from the traditional lecture-style education model and moving towards more small-group learning experiences, as well as experiential learning activities [1]. Instructional strategies such as case-based learning sessions, laboratory sessions, and earlier clinical encounters are expanding. As the COVID-19 pandemic evolved and social distancing measures were put into place, medical students faced numerous obstacles relating to their education such as the inability to gather with peers, lost sense of community, inadequate opportunities to practice crucial clinical skills, uncertainties of their roles in rotations, as well as individually grappling with virtual delivery of rigorous and difficult academic content while in quarantine [2,3]. The loss of these collective experiences poses a huge threat to the unity of the student body that medical schools and students have worked so hard to build.

There are numerous examples of schools utilizing virtual teleconferencing platforms to increase engagement during the COVID-19 pandemic [2,4]. Faculty members adjusted their lectures for both pre-clerkship and clerkship curriculum to allow for optimal delivery over an online platform. Many small-group case-based learning sessions are being facilitated online, and some schools have even been successful at providing clinical skills instructional experiences virtually [5].

The ramifications of social distancing measures and pause in medical education have also taken a toll on medical student’s psychological wellbeing. Cao et al. utilized the 7-item Generalized Anxiety Disorder Scale (GAD-7) to assess the mental health of medical students in China during the COVID-19 outbreak [6]. It was found that 24.9% of medical students experienced anxiety and of these students, 0.9%, 2.7%, and 21.3% of students experienced severe, moderate, and mild levels of anxiety, respectively. Students identified that much of their anxiety was due to stressors such as economic hardships, changes to daily life, and academic delays; whereas students who had strong social support reported lower levels of anxiety.

Medical schools have been exploring the implementation of different strategies to help cope with the mental and emotional issues faced by medical students during this pandemic. The Shiraz University of Medical Sciences developed a social media platform to offer peer mentoring services to medical students [7]. Their platform provided the opportunity for senior medical students to act as peer mentors to junior medical students and assist them in coping with the anxiety and stress brought on by the COVID-19 pandemic. Not only did students report satisfaction with the program and a significant impact on their mental wellbeing, but it also provided them with an opportunity for professional growth. 

Prior to the pandemic, maintaining a sense of support and community amongst medical students has always been a challenge faced by schools across the country. Wayne State University School of Medicine (WSU SoM) has worked at encouraging the engagement of students through the introduction of Learning Communities (LCs). Here, 300 students are divided into one of eight LCs, each with 36-42 students per class year, and each with a student elected to serve as an LC coordinator (LCC) for each medical class. Working with the LC Program Manager, LCCs take on the duty of encouraging students within his/her cohort to engage students through mentorship, social and unifying activities, scholarship, and community service. As the COVID-19 pandemic evolved, the LCCs were inspired to find creative ways to maintain connections with their peers and community members. The LC program at WSU SoM expanded their programming to increase the sense of connectedness among the student body and promote integral exposure to the realities of COVID-19 for medical students through a Virtual Conversation series. 

To our knowledge, this is the first initiative of its kind, which enabled students in the SoM to share their experiences during a pandemic while simultaneously connecting with physicians on the COVID-19 frontlines. Although many medical students have volunteered in the fight against COVID-19, they do not know what to expect in their roles as future physicians in the constantly evolving health care landscape. Herein, we discuss the topics offered through this Virtual Conversation series, and their effectiveness in promoting student engagement during the COVID-19 pandemic.

## Materials and methods

The Virtual Conversation series initiative involved LCCs individually reaching out to an array of residents and clinical physicians selected from the network of Office of Student Affairs Director of Mentoring and Student Engagement. Essential frontline physicians were handpicked based on specialty and range of experience. LCCs contacted each physician by email, with an invitation to co-host a Virtual Conversation alongside a second or third-year medical student LCC Facilitator. The physicians immediately began accepting the invitations by scheduling the Virtual Conversations over the first three-weeks of April 2020. 

Social media announcements and email invitations were then sent to the LC members by their respective LCCs. Details regarding the eight 40-minute Virtual Conversations included the name of the hosting LC and physician, conversation topic, date and time, as well as the Zoom link to join. Conversation topics included (1) utilizing telemedicine, (2) serving on the frontlines from a resident's perspective, (3) physician experience managing a pandemic versus natural disasters, (4) being an intern in the medical intensive care unit (MICU), (5) leading community resources, (6) leading and rounding, (7) an epidemiologist's perspective, and (8) unique barriers faced by homeless and lesbian, gay, bisexual, transgender and queer or questioning (LGBTQ)+ adolescents during the pandemic. 

The decision to use Zoom as the platform for the series was based on the rapid transformation to online learning and the preference to continue the uninterrupted duties and engagement of the LCs. Zoom was the chosen platform for the series as students were familiar with the interface and had regularly utilized it during the transition to remote learning through WSU SoM. A survey link was emailed to the attendees within one hour of concluding the conversations to collect the data needed to measure the series objectives. Afterwards, a composite of all the surveys was used to qualitatively analyze the data.

The Virtual Conversation series primarily aimed to inform the WSU SoM medical students who are anticipating their new roles serving on the frontlines. A Likert scale (1=extremely dissatisfied, 2=slightly dissatisfied, 3=neither satisfied/dissatisfied, 4=slightly satisfied, 5=extremely satisfied), open text, and ranking questions (extremely useful, very useful, useful, somewhat useful, not at all useful) were used to collect response. A Likert scale and ranking questions were used to standardize responses for comparison, and open text responses were included to illicit more specific feedback from attendees.

Without utilizing identifying information, evidence was gathered using the Qualtrics Survey tool to evaluate each of the presentations. WSU SoM used the survey responses to identify, measure, and appropriately address shortcomings, introduce specific COVID-19 pandemic-related curriculum, and prepare students, faculty, and staff accordingly.

## Results

The eight LCs hosted a unique student experience through the Virtual Conversation series. One hundred percent of the respondents (n=55) from each conversation of the series reported that the presentations were useful, with open text responses demonstrating 5% of students felt the topics directly covered student experiences in clinical rotations during a global health crisis, as well as student futures as physicians in the COVID-19 landscape. Students interacted with and questioned the panelists about health systems’ preparedness and vigorously changing safety protocols, the roles of medical students during the global health crisis, and more.

**Figure 1:**
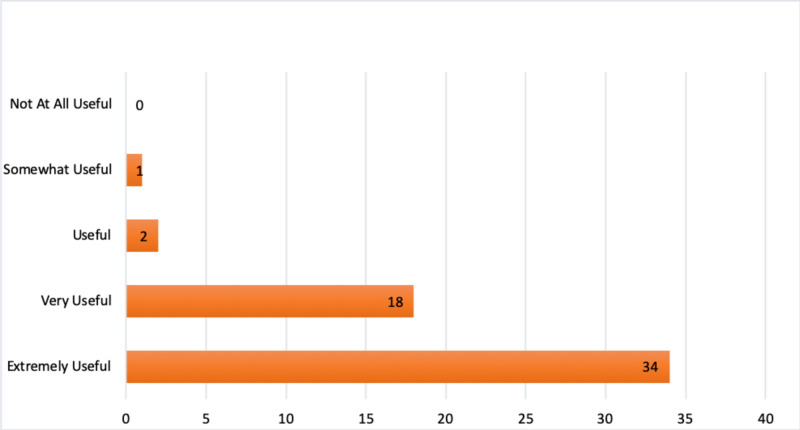
Medical students rating of usefulness of virtual conversation series

When asked to rate the usefulness of the Virtual Conversation series, 62% of medical students selected ‘extremely useful’ and 33% selected ‘very useful’. 0% of medical students selected ‘not at all useful’ (Figure 1).

**Figure 2:**
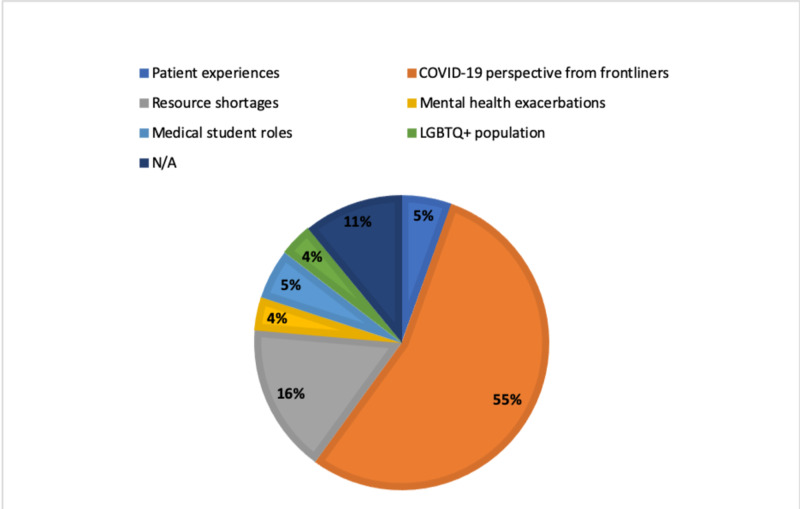
Information medical students learned from Virtual Conversation series

Qualitative text analysis was performed by two individual researchers, and any relevant discrepancies were discussed and resolved. Results of the analysis identified new information medical students learned through the Virtual Conversation series categorized according to the following: (1) reflections on patient experiences, (2) resource shortages, (3) medical student roles during the COVID-19 pandemic, (4) COVID-19 perspectives from frontliners, (5) mental health exacerbations of clinical providers during the pandemic, and (6) the impact of COVID-19 on LGBTQ+ populations. 55% of medical students reported they learned something new about the COVID-19 pandemic from the perspective of frontliners (Figure 2).

 

**Figure 3:**
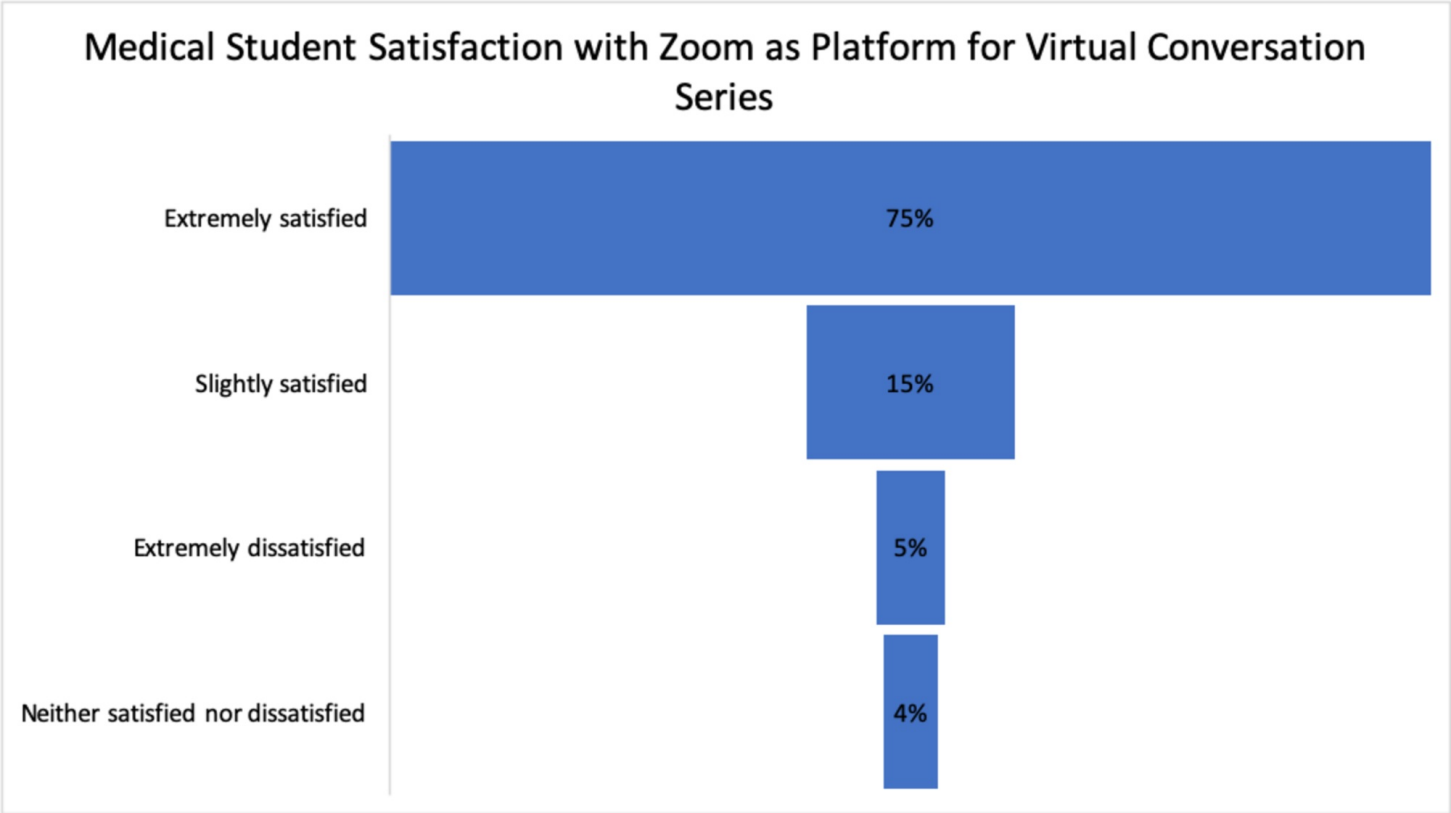
Medical student satisfaction with Zoom as platform for Virtual Conversation series

When asked, “How satisfied are you with Zoom for this type of online session?” responses indicated the use of Zoom as a platform for the series was highly received by a wide margin. Six percent of the attendees were extremely dissatisfied with the platform but did not state why. Four percent of students were neither satisfied nor dissatisfied. Contrarily, 90% of the responses ranged from slightly satisfied to extremely satisfied (Figure 3).

Students were also asked for suggestions of future topics in the interest of another installment of the Virtual Conversation series. Twenty-one students responded with suggestions among which 29% reported wanting to hear more about physicians’ perspectives from the front lines of the COVID-19 pandemic, 33% were interested in hearing more about managing COVID-19 patients, and 19% hoped to learn more about the roles of medical students and residents during this time. The remaining 19% were interested in various topics such as managing mental health as a patient care provider, the health system’s preparedness for the pandemic, and financial management as a physician.

## Discussion

The Virtual Conversation series was influenced by COVID-19 to bring awareness of the realities of the pandemic to WSU SoM LC medical students. The objective was to provide peer connectedness while educating students on the medical concerns surrounding the pandemic by using residents and physicians to engage directly with students. Conversation topics included (1) utilizing telemedicine, (2) serving on the frontlines from a resident's perspective, (3) physician experience managing a pandemic versus natural disasters, (4) being an intern in the MICU, (5) leading community resources, (6) leading and rounding, (7) an epidemiologist's perspective, and (8) unique barriers faced by homeless and LGBTQ+ adolescents during the pandemic. 

Students were asked “What did you learn about the COVID-19 pandemic that you didn’t already know?”, and two individual researchers performed qualitative text analysis to identify response themes. Results indicated topics such as mental health and burnout concerns affecting inexperienced residents, intensive care units (ICUs) quickly meeting capacity, the importance and rationale behind telemedicine, and adaptation and preparedness for pandemics are similar to that of natural disasters.

The Virtual Conversation series feedback survey was used to measure and appropriately address shortcomings, introduce specific pandemic related curriculum, and prepare students, faculty, and staff accordingly. Limitations include student sample size; although there was a total of 158 participants, only 55 students responded to the survey. However, the majority of students who responded agreed on the utility and importance of the initiative. The Virtual Conversations series allowed the WSU SoM LC program to provide an opportunity for engagement and mentorship of students while promoting and following social distancing measures. Many students were able to network with the presenting residents and physicians and proposed topics for future installments of the series. The majority of student responses indicated that future directions of the Virtual Conversation series should focus on medical student concerns and questions regarding the COVID-19 pandemic such as managing COVID-19 patients and the roles of medical students and residents during this time.

## Conclusions

The Virtual Conversation series provided a unique benefit to medical students’ exposure of the realities of COVID-19, the current landscape of healthcare, anticipation of their future roles as physicians, connectedness with their community, and opportunity to practice flexibility as they begin to apply online learning to real-world situations in the health system.  Medical students who are training to serve in their roles as frontliners have been provided with an opportunity to connect with their peers already on the frontlines, which has bolstered their preparation. With a desire for expansion of the topics presented in this Virtual Conversation series, as well as other topics that pertain to medical students’ professional development, it is recommended this initiative be incorporated into a more longitudinal component of the medical school curriculum to increase student connectedness with their peers through LCs and physician colleagues.
